# Erie County Med. Society

**Published:** 1857-02

**Authors:** 


*Erie County Med. Society.*—The annual meeting of the medical society of the county of Erie, was held in this city on Tuesday, the 13th January, 1857, continuing throughout the day. Dr. Wm. Van Pelt, the President, in the chair.

The attendance was large, and matters of much interest to the society were proposed, discussed, and acted upon; but they were generally subjects of importance only to the members of the society, and not such as to interest outsiders.

Drs. John Gilmore and G. A. Rogers, were, upon their applications, admitted to membership, "upon their compliance with the requirements of the by-laws," a form of phraseology, which many of the members, perhaps for the first time, found not to be devoid of meaning.

The receipts into the treasury for the past year had been $50.50. The expenditures $25.00.

The following officers were elected for the ensuing year:

| | |
|---|---|
| Dr. F. H. HAMILTON, - - - | *President.* |
| " JABEZ ALLEN, - - - - | *Vice-president.* |
| " JAMES M. NEWMAN, - - - | *Secretary.* |
| " C. H. WILCOX, - - - - | *Treasurer.* |
| " J. B. SAMO, - - - - | *Librarian.* |

Drs. Eastman, Hawley and Hutchins—*Primary Board.*

| | | |
|---|---|---|
| *Censors*—Dr. J. Boardman, | *Ex. in* | *Anatomy, Physiology and Surgery.* |
| " P. H. Strong, | " | *Practical Medicine and Obstetrics.* |
| " J. Barnes, | " | *Chemistry and Pharmacy.* |
| " C. C. Wyckoff, | " | *Materia Medica and Botany.* |
| " G. F. Pratt, | " | *Med. Jurisprudence and Pathology.* |

Dr. A. W. Nichols was appointed *Orator* for the semi-annual meeting in June next, and Dr. B. H. Lemons, his *substitute.*

The labors of the day were closed by the *Valedictory Address*, by Dr. Wm. Van Pelt, an article which attracted much notice and elicited strong expressions of commendation from the members present. We yield to the request of the society most cheerfully, and give it place in our Journal.

